# Practical Anthropology

**Published:** 1895-11-23

**Authors:** 


### The Study of Man.

### PRACTICAL ANTHROPOLOGY.

"On the Contact of European and Native Civilisations." A discussion held at the British Association Meeting, Ipswich, 1895, pp. 29.

The question is occasionally asked—To what practical object can the result of the study of man be applied? We gather together masses of information about every conceivable manifestation of human activity, of every grade of complexity; and we come, or hope to come, to some comprehension of the meaning and the principles which underlie them. But is there any art or practical department which stands to anthropology in the same relation as navigation to astronomy, or as agriculture to botany?

The botanist supplies the farmer with accurate information about the nature and life of plants, their relation to their circumstances, the uses to which they can be put, and the method by which existing species may be modified and improved in the quality and quantity of their produce. In a sense, in fact, he may be said to repay his debt to the plant world whence he draws his materials by the production, through the agency of the farmer, of better plants and of a better environment of plant life.

Here is, perhaps, our clue. Is it impossible to turn anthropological theory to account in our dealings with other men—to bring our knowledge of the types of civilisation which differ from our own, and of the principles which underlie them, to bear upon the modes of our own intercourse with alien races, and of our administration—which inevitably involves modification—of those which come under our rule?

Here is apparently a department, fairly to be described as practical anthropology, which might have been expected to have already made great progress among an imperial people, with so many native races and civilisations to deal with as we have. True, our unique opportunities of observation have given us a lead, though not a proportionate lead, in the field of investigation; but we shall have reason to see that as to the application of our results we find ourselves on almost virgin soil.

Few things are more striking than the apathy and ignorance displayed by all classes equally in this country in regard to the treatment of other civilisations by our emissaries, official or irresponsible. Our children are instructed indeed, some of them, in the elements of the culture of Greece and Rome; most of them in those of Judea; but of the mode of life and of thought of their Hindoo, Kaffir, or Australian fellow subjects they know as little as of the Patagonians or the Chinese. Our universities provide no regular training in this respect for our future administrators and pioneers; indeed, one of the best equipped of them has only this year rejected the proposal to make "the proper study of mankind" one of the many recognised avenues to a degree. We spend millions annually on the "conversion of the heathen," but our subscribers know for the most part, as little as they care, what the heathen are converted from, or how much or how little the process means to them. As for our colonists and adventurers, their attitude is for the most part purely and unaffectedly selfish. They are the salt of the earth, and if the native gets in their way so much the worse for the native. The political aspect of the case is the most hopeless of all. Anyone who has followed the proceedings of the House of Commons in dealing with all such questions knows the futility of entrusting the destinies of our non-European subjects to a body so typically amateur, indifferent, and uneducated in the elements of the subject. And there are episodes enough in the records of the Colonial and Indian Offices which are quite as little creditable to the tact and intelligence of their originators.

The difficulties experienced by "civilised" Governments or settlers, in dealing with races with lower or different types of culture, will be best illustrated by a few cases selected from among the more obvious.

The natives of tropical or sub-tropical climates are proverbial for the lightness of their costume, and it is probably in this particular that what is in itself an entirely natural and quite inoffensive divergence from our sub-arctic habits has most frequently given rise to the inference that nakedness has there also the same sentimental and moral associations which it has with Europeans. The Scriptural injunction to "clothe the naked" has accordingly been considered a primary obligation by many of those who have had the welfare of the native at heart, regardless, or rather in ignorance, of the fact that to dress a savage in our ill-ventilated and incommodious clothes is to subject him to discomforts and positive disorders quite as serious as those which we should experience if exposed to a tropical or arctic climate.

The change from a nomadic, or even a pastoral mode of life to a settled agricultural state, still more to a sedentary industrial career of the European type, tells in the same way very heavily on the vitality of the savage. If he does not die outright he becomes both bodily and mentally enfeebled and demoralised, and even when he is caught young and has been apparently reclaimed by years of continuous training, there appears to be no security that the "natural man" will not break loose after all, and put off in a few months the so-called "civilisation" which has never really affected him. The Australian in Charles Reade's novel is only too life-like a portrait.

Quite as disastrous is the attempt which is frequently made to impart to comparatively settled and civilised races that "elementary education" of which we are so inordinately proud. "The complex ideas of connecting forms and sounds is far too great a step for many brains; and when we succeed, to our delight, in turning out finished readers, Nature comes in with the stern reply, 'Of their children not one has been reared.'" Moreover, in a less artificial state of society than our own, the capacious and accurate memory of the native dispenses with written memoranda of facts or figures, and untrammelled social conversation with the necessity of literary study in all the ordinary circumstances of daily life. And we are, alas! only now beginning to realise, among our own people too, that the "three R's" may be faultlessly acquired without the smallest mitigation of anti-social and immoral propensities; nay, even that the very acquisition of them only gives a wider power of harm, where no higher ideal has been inculcated.

(*To be continued.*)

